# Supplementary material for: Empirical methods for controlling false positives and estimating confidence in ChIP-Seq peaks
Source: BMC Bioinformatics. 2008 Dec 5;9:523. doi: 10.1186/1471-2105-9-523 (PMC2628906; doi:10.1186/1471-2105-9-523)
Supplement: Additional File 1 — A variety of html documents from the USeq web site detailing the available applications, their best usage, output file type descriptions, command line menus, etc. [file 1471-2105-9-523-S1.zip › USeqUserGuides/JavaDoc.zip!/JavaDoc/bioroot/class-use/UserBean.html]

Uses of Class bioroot.UserBean


|  |  |  |
| --- | --- | --- |
| **Overview** \| **Package** \| **Class** \| **Use** \| **Tree** \| **Deprecated** \| **Index** \| **Help** | |  |
| PREV   NEXT | **FRAMES**    **NO FRAMES**     **All Classes** |  |


---


## **Uses of Class bioroot.UserBean**

| Packages that use UserBean | |
| --- | --- |
| **bioroot** |  |
| **bioroot.antibody** |  |
| **bioroot.oligo** |  |
| **bioroot.plasmid** |  |
| **bioroot.strain** |  |

Uses of UserBean in bioroot

| Methods in bioroot that return UserBean | |
| --- | --- |
| `UserBean` | `LoginBean.createUserBean(DBUtil bioRoot, java.lang.String host, java.lang.String IP)` |
| `static UserBean` | `Util.fetchUserBean(javax.servlet.http.HttpServletRequest request, javax.servlet.http.HttpServletResponse response)`             Sets a guest user bean if there is none. |
| `UserBean` | `UserRegistrationBean.getSubmitterUserBean()` |
| `UserBean` | `LoginBean.getUserBean()` |

| Methods in bioroot with parameters of type UserBean | |
| --- | --- |
| `static java.lang.String` | `DBUtil.buildSqlStatement(java.lang.String reagentType, UserBean user, DBUtil bioRoot, boolean descending)`             Creates a sql statement given a UserBean and a boolean as to whether to add a DESC to the order by where appropriate. |
| `static java.lang.String` | `Archive.buildSqlStatement(UserBean user, java.lang.String tableName)`             Creates a sql statement for super users and standard users returns null for everyone else, lab specific. |
| `boolean` | `DBUtil.canAlter(UserBean userBean, java.lang.String tableName, int id)`             Checks whether the user can modify or delete an item. |
| `boolean` | `ReagentBean.canModifyAll(UserBean user)` |
| `boolean` | `ReagentBean.canModifyCommon(UserBean user)` |
| `boolean` | `ReagentBean.canView(UserBean user, DBUtil bioRoot)` |
| `boolean` | `DBUtil.canView(UserBean user, java.lang.String tableName, int id)`             Checks whether the user can view an item without creating a bean, just an id |
| `java.lang.String[]` | `DBUtil.canView(UserBean user, java.lang.String tableName, java.lang.String[] ids)`             Checks whether the user can view multiple items based on id. |
| `java.lang.String[]` | `DBUtil.fetchCellTypeConcats(int id, UserBean user)`             Fetches concatinate of name and notes from CellType |
| `java.lang.String[]` | `DBUtil.fetchGeneConcat(int id, UserBean user)`             Fetches concatinate of name and notes from Gene |
| `int[]` | `DBUtil.fetchReagentCounts(UserBean user, java.lang.String tableName)`             Fetches the number of reagents in the users labgroup, and the number owned by the user. |
| `java.lang.String[]` | `Preference.getVisibilities(DBUtil bioRoot, UserBean userBean)` |
| `static java.lang.String[]` | `Util.getVisibilities(UserBean userBean, DBUtil bioRoot)`             If user is part of an organization, it returns WWW, orgName, Lab Mates, Private. |
| `static AntibodyBean[]` | `Archive.makeAntibodyBeans(DBUtil bioRoot, UserBean userBean)`             Makes an array of all visibile AntibodyBeans |
| `static java.io.File` | `CronArchive.makeArchive(DBUtil bioRoot, UserBean superUser)`             Returns null or a zip file containing a labGroup's full archive. |
| `static java.lang.String` | `Search.makeHashSelectStatement(java.lang.String table, java.util.HashMap rowScore, UserBean userBean)`             Makes an SQL SELECT statement using a HashMap. |
| `static OligoBean[]` | `Archive.makeOligoBeans(DBUtil bioRoot, UserBean userBean)`             Makes an array of all visibile OligoBeans |
| `static PlasmidBean[]` | `Archive.makePlasmidBeans(DBUtil bioRoot, UserBean userBean)`             Makes an array of all visibile PlasmidBeans |
| `static StrainBean[]` | `Archive.makeStrainBeans(DBUtil bioRoot, UserBean userBean)`             Makes an array of all visibile StrainBeans |
| `SearchHit[]` | `SearchBean.searchForSimilarMatches(java.lang.String[] columns, java.lang.String[] words, DBUtil util, boolean searchForWordBoundaryMatches, UserBean userBean)`             Searches a list of columns for a set of words, case insensitive, %word% matches. |
| `void` | `UserRegistrationBean.setSubmitterUserBean(UserBean bean)` |
| `boolean` | `OrganismBean.submitNew(DBUtil bioRoot, UserBean user)` |
| `boolean` | `MarkerBean.submitNew(DBUtil bioRoot, UserBean user)` |
| `boolean` | `GeneBean.submitNew(DBUtil bioRoot, UserBean user)` |
| `boolean` | `CellTypeBean.submitNew(DBUtil bioRoot, UserBean user)` |
| `boolean` | `DBUtil.updateCommonAccessFields(ReagentBean bean, UserBean userBean, javax.servlet.http.HttpServletRequest request)`             Just update the commonly accessible fields: lastUser, comments |
| `boolean` | `OrganismBean.updateOld(DBUtil bioRoot, UserBean user)` |
| `boolean` | `MarkerBean.updateOld(DBUtil bioRoot, UserBean user)` |
| `boolean` | `GeneBean.updateOld(DBUtil bioRoot, UserBean user)` |
| `boolean` | `CellTypeBean.updateOld(DBUtil bioRoot, UserBean user)` |

Constructors in bioroot with parameters of type UserBean

`UserRegistrationBean(UserBean user)`

Uses of UserBean in bioroot.antibody

| Methods in bioroot.antibody with parameters of type UserBean | |
| --- | --- |
| `static void` | `AntibodyBase.addPlasmid(AntibodyBean bean, java.util.HashMap nameValue, DBUtil bioRoot, UserBean userBean)` |
| `static void` | `AntibodyBase.addStrain(AntibodyBean bean, java.util.HashMap nameValue, DBUtil bioRoot, UserBean userBean)` |
| `static java.lang.String` | `AntibodyModify.deleteAntibody(AntibodyBean ab, UserBean userBean, DBUtil bioRoot)`             Attempts to delete an antibody, if sucessful, returns a "" String, otherwise an error message. |
| `static java.lang.String` | `AntibodySpreadSheet.fetchAntibodyTableRows(UserBean userBean, AntibodyBean[] beans, int[] startStop, DBUtil bioRoot, boolean checked, boolean allTabs)`             Returns table rows for every oligo according to a Preference, if allTabs==true, then all features are returned in a tab delimited format, otherwise its HTML. |
| `static java.lang.String` | `AntibodyBase.getAntibodyForm(AntibodyBean ab, DBUtil bioRoot, UserBean userBean)`             Returns an antibody form filled with the values in the bean. |
| `static java.lang.String` | `AntibodyBase.getAntibodyReport(AntibodyBean ab, DBUtil bioRoot, UserBean user, boolean commonForm)`             Returns an antibody report filled with the values in the bean. |
| `static void` | `AntibodySpreadSheet.makeAndSetAntibodyBeans(javax.servlet.http.HttpSession session, DBUtil bioRoot, UserBean userBean)`             Makes an array of AntibodyBeans based on Users AntibodyPreferences and sets them in the session. |
| `static AntibodyBean[]` | `AntibodySpreadSheet.orderAntibodies(java.lang.String featureName, UserBean userBean, AntibodyBean[] beans, DBUtil bioRoot)`             Orders antibodys, sort of nasty, can't figure another way, reflection is way too slow. |
| `static java.lang.String` | `AntibodyModify.updateAntibody(AntibodyBean bean, UserBean userBean, DBUtil bioRoot)`             Attempts to update a AntibodyBean in the database, only checks that the name is unique if it has changed. |
| `boolean` | `AntibodyBean.updateOld(DBUtil bioRoot, UserBean submitter)`             Updates a Antibody entry, don't forget to update geneId |

Uses of UserBean in bioroot.oligo

| Methods in bioroot.oligo with parameters of type UserBean | |
| --- | --- |
| `static java.lang.String` | `OligoModify.deleteOligo(OligoBean oligoBean, UserBean userBean, DBUtil bioRoot)`             Attempts to delete an oligo, if sucessful, returns a "" String, otherwise an error message. |
| `static java.lang.String` | `OligoBase.getOligoForm(OligoBean ob, DBUtil bioRoot, UserBean userBean)`             Returns an oligo form filled it with the values in the oligo bean. |
| `static java.lang.String` | `OligoBase.getOligoReport(OligoBean ob, DBUtil bioRoot, UserBean userBean, boolean commonForm)`             Returns an oligo report. |
| `static void` | `OligoSpreadSheet.makeAndSetOligoBeans(javax.servlet.http.HttpSession session, DBUtil bioRoot, UserBean userBean)`             Makes an array of OligoBeans based on Users OligoPreferences and sets them in the session. |
| `boolean` | `OligoBean.updateOld(DBUtil bioRoot, UserBean submitter)` |
| `static java.lang.String` | `OligoModify.updateOligo(OligoBean oligoBean, UserBean userBean, javax.servlet.http.HttpServletRequest request, DBUtil bioRoot)`             Attempts to update an OligoBean in the database, only checks that the name is unique if it has changed. |

Constructors in bioroot.oligo with parameters of type UserBean

`OligoBean(java.lang.String[] cells,
IdNameMatch[] genes,
IdNameMatch[] organisms,
UserBean userBean,
DBUtil bioRoot)`
  
          For bulk file upload should be 17? cells
Name, Alias Oligo Sequence, Matching Gene Sequence, uM Stock Concentration, ng/uL Stock Concentration, Volume(uL), Purification,
0 1 2 3 4 5 6 7
Modifications, Orientation, Type, Storage Location, Availibility, VisibilityBean, Handling Instructions, Target Gene Name, Organism, Notes
8 9 10 11 12 13 14 15 16 17

Uses of UserBean in bioroot.plasmid

| Methods in bioroot.plasmid with parameters of type UserBean | |
| --- | --- |
| `static java.lang.String` | `PlasmidModify.deletePlasmid(PlasmidBean plasmidBean, UserBean userBean, DBUtil bioRoot)`             Attempts to delete an plasmid, if sucessful, returns a "" String, otherwise an error message. |
| `static java.lang.String` | `PlasmidSpreadSheet.fetchPlasmidTableRows(UserBean userBean, PlasmidBean[] beans, int[] startStop, DBUtil bioRoot, boolean checked, boolean allTabs)`             Returns table rows for every oligo according to a Preference, if allTabs==true, then all features are returned in a tab delimited format, otherwise its HTML. |
| `static void` | `PlasmidBase.findOligoMatches(UserBean user, PlasmidBean[] plasmids, DBUtil bioRoot)`             Uses Blast to find oligo matches to a given array of plasmid from all the oligos in a labgroup visible to the user. |
| `static void` | `PlasmidBase.findOligos(UserBean user, PlasmidBean[] plasmids, DBUtil bioRoot)`             Uses Blast to find oligo matches to a given array of plasmid from all the oligos in a labgroup visible to the user. |
| `static void` | `PlasmidBase.findOligos(UserBean user, PlasmidBean plasmid, DBUtil bioRoot)`             Uses Blast to find oligo matches to a plasmid from all the oligos in a labgroup visible to the user. |
| `static java.lang.String` | `PlasmidBase.getPlasmidForm(PlasmidBean ob, DBUtil bioRoot, UserBean userBean)`             Returns a plasmid form filled with the values in the bean. |
| `static java.lang.String` | `PlasmidBase.getPlasmidReport(PlasmidBean pb, DBUtil bioRoot, UserBean userBean, boolean commonForm)`             Returns an plasmid report. |
| `static void` | `PlasmidSpreadSheet.makeAndSetPlasmidBeans(javax.servlet.http.HttpSession session, DBUtil bioRoot, UserBean userBean)`             Makes an array of PlasmidBeans based on Users PlasmidPreferences and sets them in the session. |
| `static PlasmidBean[]` | `PlasmidSpreadSheet.orderPlasmids(java.lang.String featureName, UserBean userBean, PlasmidBean[] beans, DBUtil bioRoot)`             Orders plasmids, sort of nasty, can't figure another way, reflection is way too slow. |
| `boolean` | `PlasmidBean.updateOld(DBUtil bioRoot, UserBean submitter)` |
| `static java.lang.String` | `PlasmidModify.updatePlasmid(PlasmidBean bean, UserBean userBean, java.util.HashMap nameValue, javax.servlet.http.HttpServletRequest request, DBUtil bioRoot)`             Attempts to update a PlasmidBean in the database, only checks that the name is unique if it has changed. |

Constructors in bioroot.plasmid with parameters of type UserBean

`PlasmidBean(java.lang.String[] cells,
IdNameMatch[] genes,
IdNameMatch[] organisms,
IdNameMatch[] markerIDMatch,
UserBean userBean,
DBUtil bioRoot)`
  
          For bulk file upload should be 16 cells
0 1 2 3 4 5 6 7 8 9 10 11 12 13 14 15 16
Name Alias Source Recipients Reference Gene Organism Markers StrainIds Location Concentration Volume Purification Availability VisibilityBean Notes Sequence

Uses of UserBean in bioroot.strain

| Methods in bioroot.strain with parameters of type UserBean | |
| --- | --- |
| `static void` | `NewStrain.addParent(StrainBean bean, java.util.HashMap nameValue, DBUtil bioRoot, UserBean userBean)` |
| `static void` | `NewStrain.addPlasmid(StrainBean bean, java.util.HashMap nameValue, DBUtil bioRoot, UserBean userBean)` |
| `static java.lang.String` | `StrainModify.deleteStrain(StrainBean strainBean, UserBean userBean, DBUtil bioRoot)`             Attempts to delete an strain, if sucessful, returns a "" String, otherwise an error message. |
| `static java.lang.String` | `StrainSpreadSheet.fetchStrainTableRows(UserBean userBean, StrainBean[] beans, int[] startStop, DBUtil bioRoot, boolean checked, boolean allTabs)`             Returns table rows for every oligo according to a Preference, if allTabs==true, then all features are returned in a tab delimited format, otherwise its HTML. |
| `static java.lang.String` | `StrainBase.getStrainForm(StrainBean sb, DBUtil bioRoot, UserBean userBean)`             Returns a strain form filled with the values in the bean. |
| `static java.lang.String` | `StrainBase.getStrainReport(StrainBean sb, DBUtil bioRoot, UserBean userBean, boolean commonForm)`             Returns an strain report. |
| `static void` | `StrainSpreadSheet.makeAndSetStrainBeans(javax.servlet.http.HttpSession session, DBUtil bioRoot, UserBean userBean)`             Makes an array of StrainBeans based on Users StrainPreferences and sets them in the session. |
| `static StrainBean[]` | `StrainSpreadSheet.orderStrains(java.lang.String featureName, UserBean userBean, StrainBean[] beans, DBUtil bioRoot)`             Orders strains, sort of nasty, can't figure another way, reflection is way too slow. |
| `boolean` | `StrainBean.updateOld(DBUtil bioRoot, UserBean submitter)`             Updates a Strain entry, don't forget to update markers and plasmids. |
| `static java.lang.String` | `StrainModify.updateStrain(StrainBean bean, UserBean userBean, java.util.HashMap nameValue, javax.servlet.http.HttpServletRequest request, DBUtil bioRoot)`             Attempts to update a StrainBean in the database, only checks that the name is unique if it has changed. |
| `static boolean` | `StrainModify.updateStrainCommonAccessFields(StrainBean bean, UserBean userBean, java.util.HashMap nameValue, DBUtil bioRoot)`             Just update the commonly accessible fields: restock, lastUser, remainingAliquots, comments |

Constructors in bioroot.strain with parameters of type UserBean

`StrainBean(java.lang.String[] cells,
IdNameMatch[] cellTypes,
IdNameMatch[] organisms,
IdNameMatch[] markerIDMatch,
UserBean userBean,
DBUtil bioRoot)`
  
          For bulk file upload should be 23 cells
0 Name
1 Alias
2 Source
4 Reference
5 Type Bacteria, Yeast, Worm, Insect, Plant, Fish, Cell Line, Bird, Mammal or Other
6 Recipients
7 Organism name
8 Markers names, comma seperated
9 Plasmid Ids
10 Parent Strain Ids
11 Storage Location
12 Mutagen EMS, ENU, MMS, MNU, X-ray, UV, Transposon, T-DNA, Spontaneous or Other
13 Genotype/ Background
14 Phenotype
15 Growth Conditions
16 Restock
17 Remaining Aliquots
18 Array Type Extra Chromosomal or Integration
19 Seed Type Individual Line, Individual Pool, Set of Lines, or Set of Pools
20 Cell Type (Cell Lines)
21 Passage Number ( Cell Lines)
22 Mating Type ( Yeast) diploid, a, alpha, h+, or h-)
23 Availability
24 VisibilityBean
25 Notes

---


|  |  |  |
| --- | --- | --- |
| **Overview** \| **Package** \| **Class** \| **Use** \| **Tree** \| **Deprecated** \| **Index** \| **Help** | |  |
| PREV   NEXT | **FRAMES**    **NO FRAMES**     **All Classes** |  |


---
